# Molecular analysis of Annexin expression in cancer

**DOI:** 10.1186/s12885-022-10075-8

**Published:** 2022-09-19

**Authors:** Tobias Hein, Peter H. Krammer, Heiko Weyd

**Affiliations:** 1 Division of Immunogenetics, Tumour Immunology Program, German Cancer Research Centre, 69120 Heidelberg, Germany; 2 Faculty of Biosciences, Ruprecht-Karls-University Heidelberg, 69120 Heidelberg, Germany

**Keywords:** Annexins, Apoptotic cells, Tumour microenvironment, Peripheral tolerance, Immunosuppression

## Abstract

**Background:**

Uptake of apoptotic cells induces a tolerogenic phenotype in phagocytes and promotes peripheral tolerance. The highly conserved Annexin core domain, present in all members of the Annexin family, becomes exposed on the apoptotic cell-surface and triggers tolerogenic signalling in phagocytes *via* the Dectin-1 receptor. Consequently, Annexins exposed on tumour cells upon cell death are expected to induce tolerance towards tumour antigens, inhibiting tumour rejection.

**Methods:**

Expression analysis for all Annexin family members was conducted in cancer cell lines of diverse origins. Presentation of Annexins on the cell surface during apoptosis of cancer cell lines was investigated using surface washes and immunoblotting. Expression data from the GEO database was analysed to compare Annexin levels between malignant and healthy tissue.

**Results:**

Six Annexins at least were consistently detected on mRNA and protein level for each investigated cell line. AnxA1, AnxA2 and AnxA5 constituted the major part of total Annexin expression. All expressed Annexins translocated to the cell surface upon apoptosis induction in all cell lines. Human expression data indicate a correlation between immune infiltration and overall Annexin expression in malignant compared to healthy tissue.

**Conclusions:**

This study is the first comprehensive analysis of expression, distribution and presentation of Annexins in cancer.

**Supplementary Information:**

The online version contains supplementary material available at 10.1186/s12885-022-10075-8.

## Background

Annexins (Anxs) are an evolutionarily conserved protein superfamily. The human Anx family is composed of 12 members, AnxA1 to AnxA13 (AnxA12 being a pseudogene), with each member consisting of a C-terminal Anx core domain as well as a flexible N-terminus [1]. The core domain is evolutionary conserved throughout the family with respect to its secondary and tertiary structure whereas the N-terminus is unique to each family member, varies in sequence and length and is the site where most post-translational modifications occur [2, 3]. Expression of Anxs is ubiquitous for all cells in the body. While most human Anxs, such as AnxA1, AnxA2, AnxA4-7 and AnxA11, are widely expressed, others are more tissue-restricted, like AnxA9 in the tongue, AnxA10 in the stomach and AnxA13 in the small intestine [4]. Anxs typically reside in the cytosol but upon induction of apoptosis, cytosolic Anxs (as shown for AnxA1, AnxA5 and AnxA13) translocate to the cell surface [5–8]. Here, the Anx core domain remains attached to negatively charged membrane lipids, such as phosphatidyl serine (PS), *via* Ca^2+^ ion bridging [5]. Upon externalisation of AnxA1 during apoptosis, the N-terminus is expelled from the core region, becomes accessible for proteolytic cleavage and is released into the extracellular space, where it acts as a chemotactic stimulus [9–11].

Work from our group and others has demonstrated that AnxA1, AnxA5 and AnxA13, as well as the isolated AnxA1 core domain, similar to apoptotic cells (ACs), prevent the activation of dendritic cells (DCs) and macrophages (MΦs) by inhibition of inflammatory cytokine secretion and reduced expression of co-stimulatory surface molecules [7, 12–14]. This way, exposed Anxs mediate immunosuppressive effects of ACs on phagocytes and contribute to peripheral tolerance [6, 7]. Central to Anx-mediated signalling is the highly conserved Anx core domain, present in all Anx family members. Accordingly, we could show that the tolerogenic function is redundant for AnxA1, AnxA5 and AnxA13, and thus, most likely, throughout most members of the Anx family [7].

An important aspect of this tolerogenic mechanism is the implication that Anxs on apoptotic cancer cells, which occur in the steady-state tumour and, increasingly, after chemotherapy, may also induce tolerance towards tumour antigens associated with apoptotic cancer cells. Thus, exposure of Anxs on apoptotic cancer cells may facilitate immune evasion and inhibit tumour immune rejection [6].

Cell death in malignant disease is an essential component of tumour dynamics and most tumours are associated with a substantial loss of cells [15]. Due to this "cell loss factor" tumours as a whole are often found to grow more slowly than the proliferation rate of single tumour cells might suggest [16]. However, rapid growth rates are rare and evolving tumours are characterised by a dynamic interplay between cell proliferation and cell death [17]. Mechanistically, apoptosis-inducing stimuli, such as hypoxia or growth factor starvation, are common in rapidly growing tumour-cell populations. Moreover, multiple pathways exist by which activated oncogenes induce apoptosis [18–21]. Given the properties of ACs and their capacity to modulate phagocytes such as MΦs and DCs, apoptotic tumour cells likely provide cues inducing an immunosuppressive tumour microenvironment [22]. Along the same lines, different studies on human tumours pointed out a correlation between reduced apoptosis due to high BCl-2 expression and a favourable prognosis [23–27]. Accordingly, apoptotic indices show a strong association between high-grade malignancies and a high rate of apoptosis [28]. Moreover, prominent apoptosis is suggestive of an adverse prognosis, as has been found, for example, in several classes of Non-Hodgkin’s lymphoma, colorectal cancer (CRC), breast cancer, endometrial carcinoma and bladder cancer [28–32]. The immunosuppressive properties of ACs have been shown to be critical in dampening adaptive anti-tumour immunity through interactions of ACs with intra-tumoural phagocytes, such as MΦs and DCs. Apoptosis of tumour cells contributes to oncogenesis, for example, through recruitment and activation of tumour-associated MΦs (TAMs) that, upon interaction with ACs, become polarised towards an M2 anti-inflammatory and tolerogenic phenotype [33, 34]. In addition to the inherent apoptosis prevalent in growing tumours [35], most current cancer therapies aim to induce an increase in cancer cell death. For the majority of classical therapies, like chemotherapeutic drugs and radiation, the mode of cell death elicited by the treatment is the induction of apoptosis rather than primary necrosis [36–38].

As mentioned above, the immunosuppressive activity of Anxs, mediated by the conserved core domain, is likely a redundant feature throughout all Anxs. Therefore, this study aimed to investigate and characterise the expression of all Anxs in various cancer entities and their translocation to the cell surface during apoptosis. In all tissues analysed several Anxs (6-10) were expressed to different extents, and all of the Anxs present in the cytosol were readily externalised upon apoptosis induction. Moreover, data from online databases indicated that Anx expression levels correlated with tumour immune status. Thus, this comprehensive analysis of Anx expression in cancer provides further insight into this novel class of immune suppressive molecules, which may become relevant for future anti-tumour treatment strategies.

## Methods

### Cell culture

All cell lines were maintained at 37 °C in Dulbecco’s Modified Eagles Medium (DMEM) or RPMI-1640 (both Sigma-Aldrich) supplemented with 10 % (v/v) heat-inactivated foetal calf serum (FCS). All used cell lines were authenticated by genotyping and all experiments were performed with certified mycoplasma-free cells. Details about used cell lines are given in Supplementary Information Table S1.

### Isolation of human cells

Preparation of Monocyte-derived human DCs was conducted as described previously [6].

For the preparation of neutrophils, blood was separated using Ficoll solution and the pellet was thoroughly resuspended in 50 ml 1 % Dextran/PBS. The solution was incubated 30 min at RT and the supernatant harvested carefully and centrifuged 10 min at 500 x g at 4 °C. In order to lyse erythrocytes in the resulting pellet, it was resuspended for 1 min in 5 ml ice-cold 0.2 % NaCl before adding 5 ml ice-cold 1.6 % NaCl and 30 ml PBS. Finally, the solution was centrifuged for 15 min at 200 x g at 4 °C and the neutrophil pellet resuspended in medium and either cultured for 2 d in 6-well plates at 2 x 10^6^/ml or used directly. For the EDTA surface washes, 20 - 30 x 10^6^ neutrophils were taken.

### Apoptosis induction **and cell death analysis**

Early apoptotic 624.38 Mel cells were generated by irradiation with 500 mJ/cm^2^ UV-C. Irradiated cells were incubated at least 16 h before they were used in further assays. For the chemical induction of apoptosis in cancer cell lines, cells were treated with either 50 μM Etoposide (Cayman Chemical) or 100 μM Dimethylfumarate (DMF, Sigma-Aldrich) [39] or left untreated as a control. After resting for 24 to 48 h, cells were detached using Accutase solution (Biolegend), centrifuged and resuspended in Anx Binding Buffer (10 mM HEPES, 140 mM NaCl, 2.5 mM CaCl_2_ in H_2_O). Neutrophils become apoptotic after 2 d of culture and were compared with freshly isolated neutrophils. Cell viability was determined by staining with AnxA5-FITC (Immunotools) and 7AAD (Sigma-Aldrich) according to the manufacturer's instructions and subsequent analysis in flow cytometry.

### EDTA surface wash and cytokine suppression assay

Apoptotic and control cells were washed twice with cold Anx Binding Buffer, resuspended in ice-cold 20 mM ETDA/PBS and incubated for 3 min on ice to yield the calcium-dependent surface-bound Anxs. After centrifugation, supernatant was used for cytokine suppression assays and immunoblotting.

The cytokine suppression assay was conducted as described previously [6]. Enzyme Linked Immunosorbent Assay (ELISA) was performed using BD OptEIA kits according to manufacturer’s protocol. Cytokine secretion is presented normalised to stimulation minus background (((treated - untreated) / (stimulation only - untreated)) *100).

### Quantitative reverse transcription and real-time PCR (qPCR)

Total mRNA was isolated from cells using the RNeasy Maxi Kit (Qiagen) according to the manufacturer’s instructions. Residual DNA was removed through on-column DNAse digestion. AMV reverse transcriptase (New England Biolabs) was used to generate cDNA which was in turn quantified by detection of SYBR Green Power incorporation (SYBR Green PCR Master Mix, Sigma-Aldrich) using the ABI Prism 7500 sequence detector system (Applied Biosystems). The housekeeping gene GAPDH was used as an internal control. All primers were synthesised by Sigma Aldrich. Reactions were performed in triplicate to account for technical variation. Quantification of relative gene expression levels was accomplished using the ΔΔC_T_ method. Detailed sequences of the primers used are listed in Supplementary Information Table S2.

### SDS-PAGE and immunoblot

Proteins were separated using SDS-PAGE and then transferred onto a nitrocellulose membrane using a wet blot-transfer system. Membranes were blocked with 5 % milk powder in TBS-T for at least 1 h at RT. For protein detection, membranes were incubated with primary antibodies (diluted in TBS-T/1 % BSA/N_3_) overnight at 4 °C or for 1 h at RT. The next day, membranes were washed 3 times with TBS-T and incubated for 1 h with a horseradish peroxidase (HRP)-conjugated secondary antibody at RT. Finally, membranes were washed 3 more times with TBS-T, developed using Western Lightning Plus-ECL detection solution (Perkin Elmer) and recorded with the Chemi-Smart 5100 System. If further antibodies were to be applied to the same membrane, the HRP was first inactivated by three incubation steps in 1 M glycine, pH 1.8. The membrane was then neutralised with TBS-T and blocked again before incubation with the next antibody. Details about the primary and secondary antibodies used are listed in Supplementary Information Tables S3 and S4, respectively.

### Databases

Data of Anx mRNA expression in healthy tumour-adjacent and malignant tissue was taken from the Gene Expression Omnibus (GEO) database (https://www.ncbi.nlm.nih.gov/geo/) from data sets listed in Supplementary Information Table S5. The expression levels of all members of the Anx family were added and compared between samples from the indicated cancer entities. The number of available patient samples ranged from 7 to 62 per entity. Except for breast cancer data, all datasets provided paired data for each patient. Microarray expression data is normalised using the MAS5 method (breast adenocarcinoma, renal clear cell carcinoma (RCC), prostate carcinoma) or the RMA method (colorectal carcinoma (CRC), oesophagus carcinoma, non-small cell lung carcinoma (NSCLC), pancreatic ductal-adenocarcinoma (PDAC)).

Data of CLEC7A mRNA expression in healthy tumour-adjacent and malignant tissue was taken from the TMNplot.com Analysis Platform. All datasets provided 70 to 210 paired data sets for each entity. Survival data of patients was taken from the Human Protein Atlas. Patients were stratified into high and low CLEC7A expressing groups based on mRNA expression data from The Cancer Genome Atlas.

### Statistical analysis

Results are shown as mean ± standard deviation or as a box plot with the median highlighted. Statistical significance was analysed using a two-sided Student's *t*-test using the GraphPad Prism software (SCR_002798). Results with *p* values below 0.05 were considered statistically significant.

## Results

### Expression profiles of the Anx family members

Apoptotic tumour cells are engulfed and processed by phagocytes within the tumour microenvironment [40]. Our previous results have shown that several Anx family members, when exposed upon apoptosis induction, constitute a redundant, immune suppressive and tolerogenic signal towards engulfing phagocytes [3, 7]. Anxs, which may be present to variable degrees within tumour cells and externalised during tumour cell apoptosis, could, therefore, induce tolerance towards antigens present within those cancer cells and inhibit tumour rejection [6]. Although expression data of single Anxs in different tissues, cell lines and cancer entities is readily available [41–43], to our knowledge, an expression analysis of the Anx family as a whole in tissues or cell lines has not been performed so far. Due to redundancy of the Anx core domains as mentioned above, we evaluated the total as well as the individual expression of Anx family members in different tumour entities, as downregulation of one Anx could be compensated by upregulation of another.

**Fig. 1:**
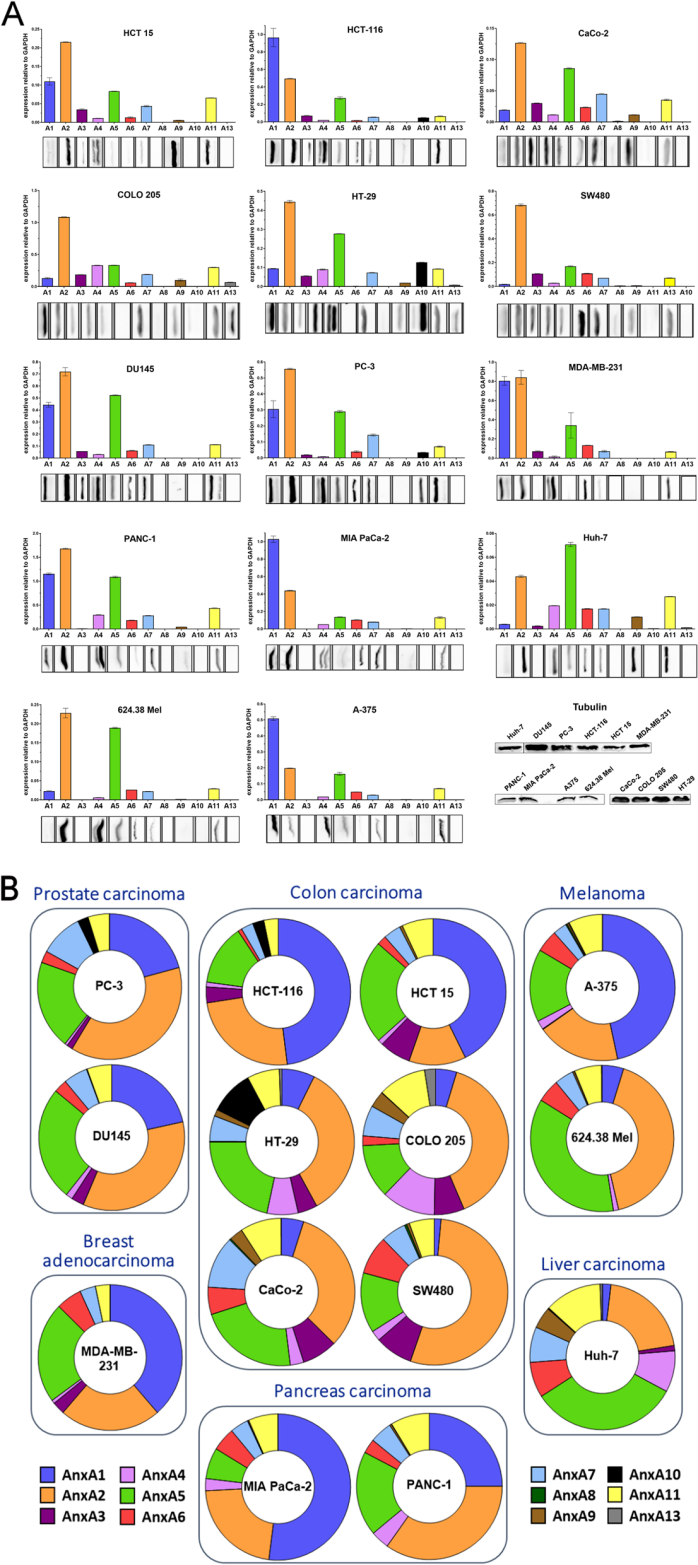
Representative Anx fingerprints on mRNA and protein level for all investigated cell lines. **A** The mRNA expression levels of all Anx family members (A1 – A13) in all tested cell lines were detected *via* RT-qPCR and depicted relative to GAPDH expression (bar diagrams). The qPCR data was confirmed on protein level using immunoblot shown below the bar diagrams, using 50 μg total protein/ lane. The corresponding Tubulin bands are shown in the bottom right corner. Full-length blots are supplied in Supplementary Information Figures S4, S5, S6. Error bars represent mean ± s.d. of technical triplicates. **B** The expression of each Annexin (Anx) family member depicted as a fraction of the total Anx expression in all tested cancer cell lines. Cancer tissues of origin for the cell lines are indicated.

As a model system, the expression of all members of the Anx family for 14 different cell lines derived from 6 cancer entities has been examined *via* RT-qPCR to create an "Anx fingerprint", the individual Anx expression profile of a tissue or cell line [3]. As presented in Figure 1, we were able to detect expression of each of the 12 Anxs in at least one of the cell lines analysed. Moreover, each cell line expressed 6-10 different Anxs, with high expression levels of at least 2 different members. To assess whether mRNA expression is reflected in the actual protein content, we analysed the individual Anx protein level in every cell line *via* immunoblot of cellular lysates. As can be seen in the lower panels of Figure 1A, Anx protein bands correlated very well with Anx mRNA signals. Importantly, the stability of Anx expression was tested over the course of several months of cell culture. After 6 to 8 months of culture, expression of all Anxs in all investigated cell lines showed remarkably little fluctuation, resulting in highly similar Anx expression profiles (Supplementary Information Figure S1). The expression of each Anx family member, therefore, proved to be very stable in these model cancer cell lines.

Given this stability of expression, we compared tumour cell lines of the same tumour entity. However, the Anx fingerprints of cell lines from one tumour yielded no consistent pattern. On the contrary, major differences in the amount and distribution of Anxs were detected, not only between cell lines derived from different cancer entities but also between cell lines from the same type of cancer, and Anx fingerprints could not be utilised to distinguish cancer entities from each other (Figure 1B).

In general, in all tested cell lines, 5 – 8 Anx family members were expressed to a medium or high degree. Most prominently, AnxA1, AnxA2, as well as AnxA5 constituted the major part of the overall Anx expression, with these three accounting for up to 86 % of total Anx expression (Figure 1B). In contrast, AnxA8 and AnxA13 could only be detected in very low levels in one and two of the tested cell lines, respectively. With respect to the redundant function of Anxs as mentioned above, a high expression level of at least one Anx was detectable in all cell lines indicating the possibility of a suppressive function upon externalisation on apoptotic tumour cells.

### Detection of Anxs on the surface of apoptotic cancer cells

**Fig. 2:**
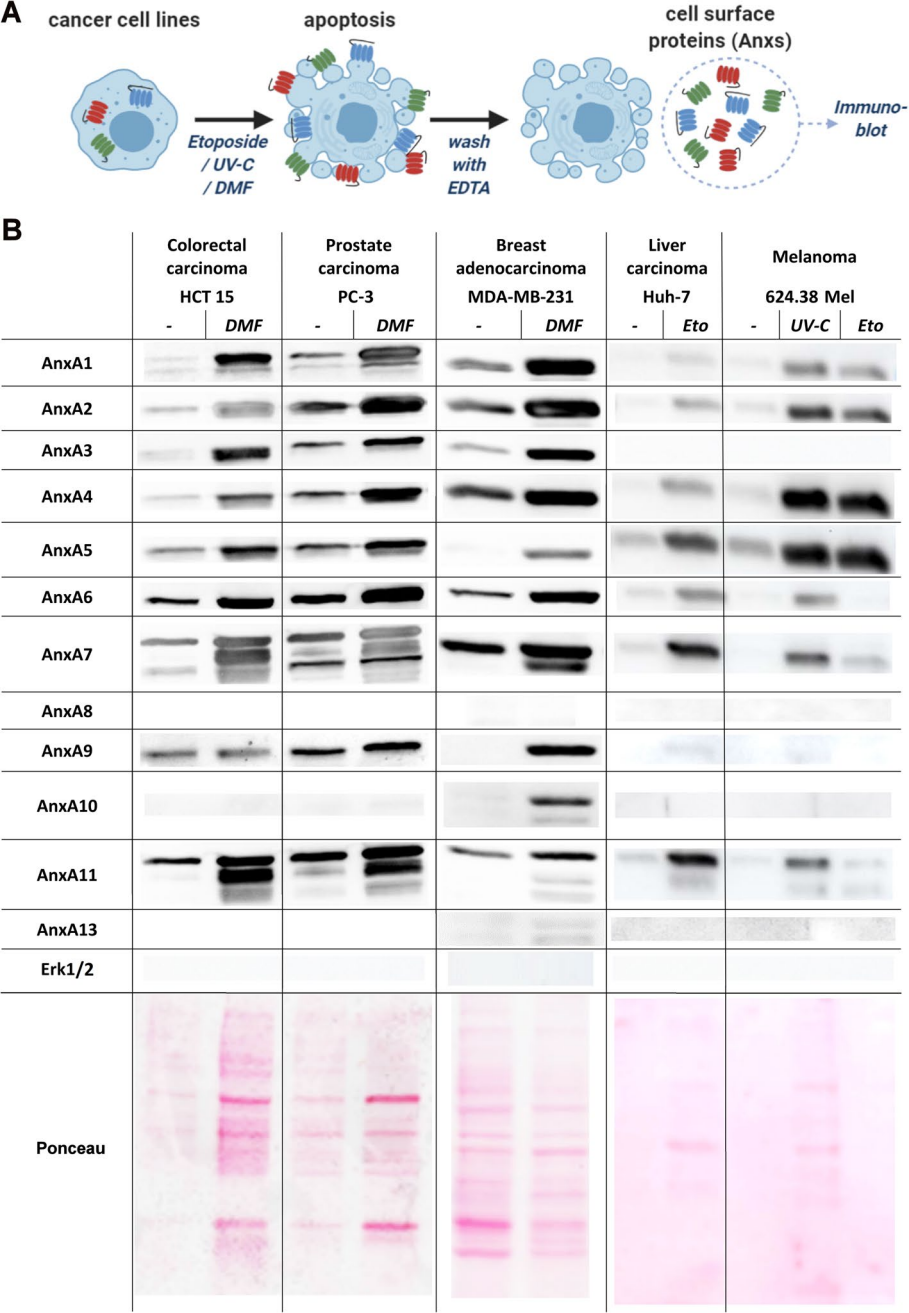
Anx family members translocate to the surface of apoptotic cancer cells. **A** Schematic representation of experimental procedure. Apoptosis in cells was induced by treatment with Etoposide (Eto), Dimethylfumarate (DMF) or UV-C radiation followed by incubation for 48 h. Subsequently, the cell surface was washed with 20 mM EDTA/PBS and supernatants were analysed by immunoblot. Washes of the same number of untreated live cells served as controls (-). **B** EDTA washes of indicated cancer cell lines before and after apoptosis induction were probed for Annexin family members (AnxA1-A13) by specific antibodies. Detection of cytosolic Erk1/2 was used as a control for cell membrane integrity. General protein content of the washes was indicated by Ponceau staining. Full-length blots are supplied in Supplementary Information Figures S7, S8, S9. In all experiments, the induction of apoptosis was monitored and confirmed by flow cytometry using AnxA5/7AAD staining (see Supplementary Information Figure S3).

Induction of apoptosis has been shown to prompt the translocation of several Anxs to the outer cell membrane where they remain bound to negatively charged Phospholipids in a Calcium-dependent manner [6, 7, 44, 45]. Thus, Anx externalisation on the surface of apoptotic cells can be analysed by washing cells in EDTA-containing buffer [7] (Figure 2A). These washes, which contain high amounts of Anxs [6], suppress the activation of human DC significantly (Supplementary Information Figure S2A-C). Interestingly, we also observed an immune-suppressive effect of surface washes from apoptotic tumour cells (Supplementary Information Figure S2D). Yet, translocation of immunosuppressive Anxs has not been demonstrated in a comprehensive way for all expressed Anx family members during apoptosis of cancer cells. In order to investigate apoptotic Anx externalisation, cell lines derived from different types of cancer were treated with Etoposide [38], UV-C radiation or dimethyl fumarate (DMF) [39, 46] to induce apoptosis. Flow cytometric analysis of apoptotic rates in these cell lines can be seen in Supplementary Information Figure S3. Subsequently, apoptotic cells were washed with EDTA to remove all calcium-bound Anxs from the cell surface (Figure 2A) [6, 7]. These EDTA-washes were then tested for the presence of all Anx family members *via* immunoblot and compared to EDTA-washes of live cells. As can be seen in Figure 2B, the amount of each Anx - if expressed in the cells (please refer to Figure 1A) - was strongly increased in EDTA washes from apoptotic cells compared to EDTA-washes of live cells. The cytosolic protein Erk1/2 was not detected in the EDTA wash fraction, confirming that cytosolic proteins were not extracted passively from cells due to the preparation procedure. Different methods of apoptosis induction applied to the same cell line led to similar exposure of Anxs on the cell surface (Figure 2 and data not shown). Thus, these experiments demonstrate that externalisation on apoptotic tumour cells is a general feature of the Anx family as a whole, independent of the mode of apoptosis induction and origin of cancer cell lines. We also investigated if Anxs could be detected in apoptotic cell culture supernatants, which would imply the secretion of Anxs. In the presence of extracellular amounts of calcium (>1mM), all annexins show a very high affinity (in the low nM range) to negatively charged phospholipids [2]. Due to this fact, it might be rightly assumed that any annexin in solution will be bound to membranes of, e.g., exosomes, microvesicles or apoptotic bodies and cell remnants [47, 48]. To this aim, we analysed culture supernatants of 3 different cancer cell lines (624.38 Mel, MDA-MB-231 and HCT 15) exemplarily for the presence of Annexin A1. We did not detect Annexin A1 in culture supernatants of untreated, live tumour cells. Upon apoptosis induction, however, we detected minute amounts of Annexin A1 in supernatants of the cancer cell line HCT 15 only (data not shown). We did not determine if this Annexin was lipid/membrane bound or indeed soluble and conclude that secretion of Annexin A1 is not a general characteristic of apoptotic tumour cell death under our experimental conditions.

### Anx expression levels in cancer and healthy tissue

**Fig. 3:**
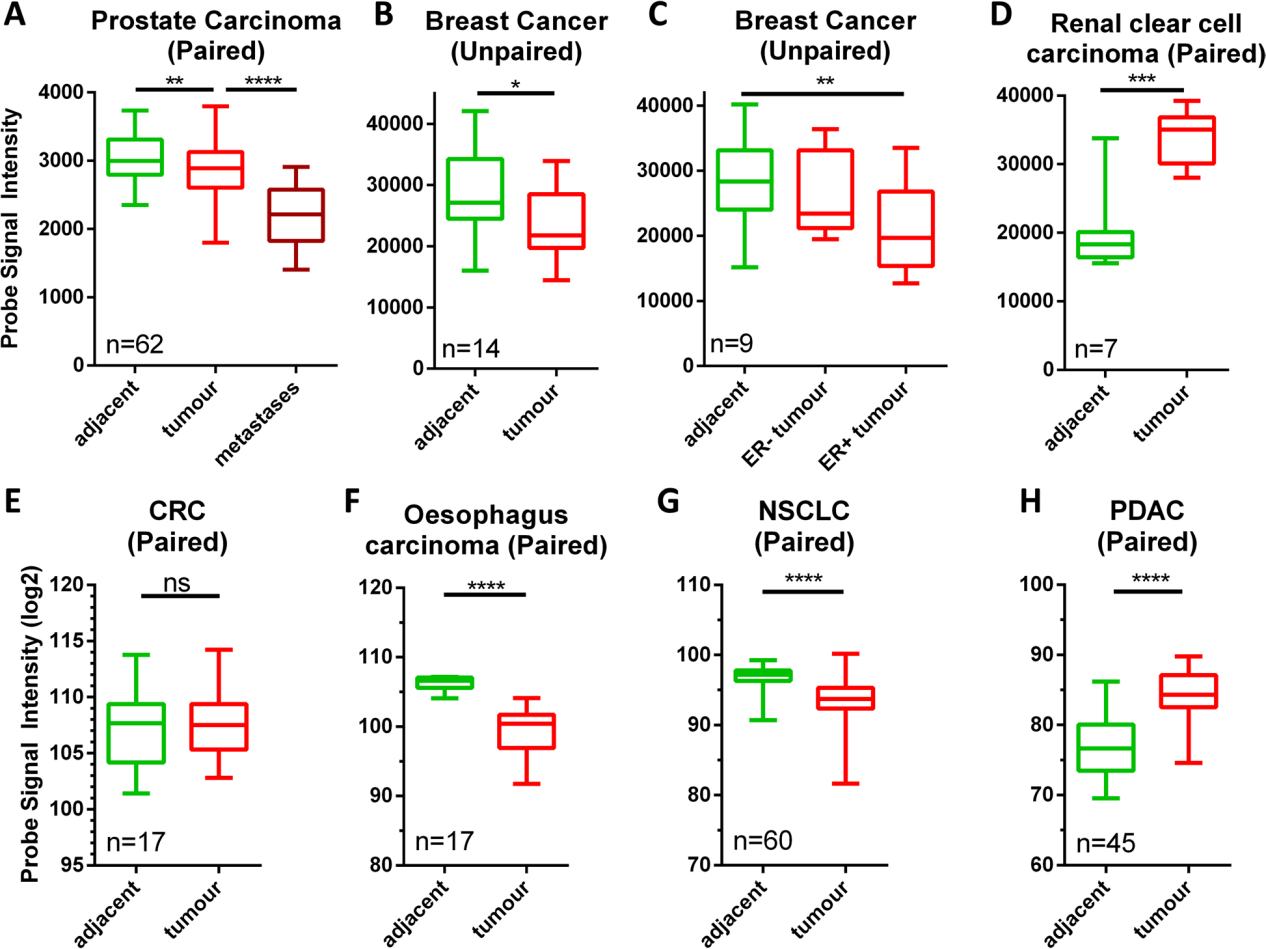
Comparison of Anx expression between healthy and malignant tissue. Data of Anx mRNA expression in healthy tumour-adjacent and malignant tissue was taken from the Gene Expression Omnibus (GEO) database. The expression levels of all 12 members of the Anx family were added and compared between samples from the indicated cancer entities. The number of available patient samples is indicated in the corner of each graph. Except for breast cancer data (**B**, **C**), all datasets provided paired data for each patient. Microarray expression data is normalised using the MAS5 method (**A** - **D**) or the RMA method (**E** – **H**). Significance was calculated using a paired (unpaired for breast cancer) two-tailed t-test (**** *p*<0.0001, *** *p*<0.001, ** *p*<0.01, * *p*<0.05, ns not significant).

**Fig. 4:**
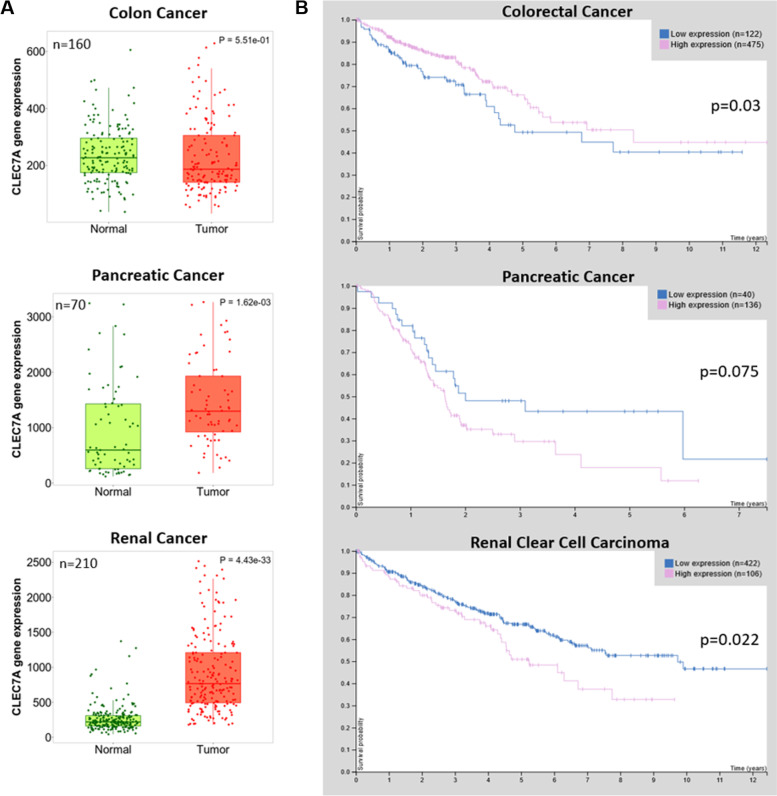
Survival rate correlates with increased Anx receptor CLEC7A expression in cancer entities with increased total Anx expression. **A** Data of CLEC7A mRNA expression in healthy tumour-adjacent and malignant tissue was taken from the TMNplot.com Analysis Platform. All datasets provided paired data for each patient. The number of available patient samples is indicated in the corner of each graph, as is the *p* value which was calculated using a paired Wilcoxon statistical test. **B** Survival data of patients suffering from the indicated malignancies taken from the Human Protein Atlas. Patients were stratified into high and low CLEC7A expressing groups based on mRNA expression data from The Cancer Genome Atlas. The number of available patient samples is indicated in the corner of each graph, as is the log-rank *p* value for the Kaplan-Meier plot showing results from analysis of correlation between mRNA expression level and patient survival.

Data presented above show well detectable expression and externalisation of Anxs in all cancer cell lines tested. Given the tolerogenic function of the Anx core domain [7, 12, 14], a high level of overall Anx expression might, thus, convey a selective advantage in tumours sensitive to rejection mediated by the immune system. Therefore, we investigated relative differences in Anx mRNA expression between healthy and malignant human tissue *in silico*. For this purpose, the NCBI Gene Expression Omnibus (GEO) supplied 8 complete data sets of expression levels for the Anx family from 7 cancer entities (Figure 3). The numbers of patients per data set range from 7 to 62, each providing paired (except for breast cancer) samples taken from the tumour as well as from tumour-adjacent healthy tissue. In order to compare overall expression of the whole Anx family, expression values of all 12 Anx members were added together for each sample. In 4 of the 7 tumour entities (prostate carcinoma, breast adenocarcinoma, oesophagus carcinoma and non-small cell lung carcinoma (NSCLC)), overall Anx expression was significantly reduced in malignant samples (Figure 3 A-C, F, G). Where metastasis data was available (prostate cancer), Anx expression was even further reduced in these samples. In contrast, Anx expression in the renal clear cell carcinoma (RCC) and pancreatic ductal-adenocarcinoma (PDAC) data was significantly increased after malignant transformation (Figure 3 D, H). In CRC, total Anx expression did not differ between healthy and malignant samples (Figure 3 E). These data indicate a heterogeneous situation, where overall Anx mRNA expression after transformation is not consistently regulated but appears to be dependent on the cancer entity. Interestingly, we detected that certain tumour entities, which show increased Annexin expression correlated with immune infiltration (RCC, PDAC [89,90]), also show a clear correlation of high Annexin receptor expression with worse prognosis (Figure 4) [14, 49, 50]. While in CRC, which did not show increased Anx expression upon malignant transformation, this correlation is not seen in the respective survival curves. This might indicate that tumours expressing high amounts of Annexin as well as the tolerogenic Annexin receptor cannot be controlled by the immune system efficiently.

Taken together, the evidence provided in our study shows that several members of the Anx family are well and stably expressed by every cancer cell line analysed. Moreover, externalisation of all Anxs expressed in a given cancer cell line was clearly detectable upon apoptosis induction. However, clinical relevance of this expression with respect to induction of peripheral tolerance and immune evasion will be dependent on various factors such as immune cell infiltration and expression of tumour antigens.

## Discussion

On the surface of apoptotic cells, several Anxs trigger tolerogenic signalling in phagocytes *via* binding of their highly conserved core domain to the Dectin-1 receptor on phagocytes [14]. Thereby, Anxs mediate immunosuppressive effects of apoptotic cells on DCs and induction of peripheral tolerance against self antigens [6, 7, 14]. Consequently, Anxs on apoptotic cancer cells may also induce tolerance towards tumour antigens, inhibiting tumour rejection [6, 51]. To gain insight into regulation of this Anx checkpoint system [51], we analysed the presence and distribution of all Anx family members as well as their translocation to the apoptotic cell surface using various cancer cell lines.

Anx expression profiles from qPCR data of 14 cell lines derived from 6 different types of cancer were compiled into Anx fingerprints and validated on protein level *via* immunoblot. The expression profiles proved stable in these cell lines over the course of several months of cell culture. Thus, we propose that these data provide a valid and reproducible overview of expression and ratio of Anxs in cancer tissues. *In vivo*, however, depending on the type, growth rate and mutational load of the tumour, Anx expression might be subject to major changes over time. Such changes might be expected upon, *e.g*., dedifferentiation of tumour cells or regulation of Anx expression during development and cellular differentiation [52, 53]. Moreover, not being as homogenous as cell lines, expression of Anxs in tumours at any given time point is likely to differ locally within tumour tissue as well [54–56].

Possibly due to these changes in expression as discussed above or due to the heterogeneous nature of cancer, no cell lines shared identical Anx profiles, even within the same cancer entity. In all cell lines, 6 to 8 Anxs were expressed to a medium or high degree, with AnxA2, AnxA5 and AnxA1 contributing the major fraction of total Anx expression. These 3 Anxs are also the most investigated family members and are implicated in several essential cellular processes, such as proliferation, adhesion, motility, angiogenesis, endocytosis, membrane repair, cell cycle regulation, apoptosis, signal transduction, as well as the regulation of metabolic enzymes [57–61]. With respect to malignancies, AnxA2 is expressed abundantly in various types of tumours where it is described to play a critical role in epithelial-mesenchymal transition [62, 63], cytoskeleton dynamics and tumour cell motility [64, 65], to regulate cell cycle and proliferation [66, 67] and to promote tumour angiogenesis [68, 69]. Similarly, dysregulated expression of AnxA5 has been implicated in the development, progression, metastasis, treatment and prognosis of a variety of cancers [70–72]. An increasing number of studies have shown the expression and function of AnxA1 in tumours to be tissue- and tumour-specific [43, 73]. Upregulation of AnxA1 was observed in breast cancer, hepatocellular carcinoma and melanoma [74–76], whereas downregulation was observed in gastric, prostate and oral cancer [60, 77, 78]. These reports on Anxs A1, A2 and A5 illustrate the magnitude and diversity of effects specific Anx family members may exert on tumour development. For the most part, however, only data of isolated Anxs have been analysed in cancer, and most of the effects mentioned above refer to intracellular functions of Anxs. An extracellular and immunoregulatory role has been noted only in few instances [79, 80], particularly for the AnxA1 N-terminus [81, 82]. In addition, Anxs are known to be secreted into the extracellular space associated with extracellular vesicles [83, 84]. This way, Anxs have been shown to play an important role in the development of tumours, *e.g.* by participating in epithelial-mesenchymal transition, regulating immune cell behaviour, and promoting neovascularisation [85].

The influence of Anxs on phagocytes relies on translocation of cytosolic Anxs to the outer leaflet of the plasma membrane during apoptosis. It was previously shown that AnxA1, AnxA2, AnxA4, AnxA5 and AnxA13 are externalised in the early phases of apoptosis [6, 7, 10]. However, particular Anxs were investigated one at a time only. Now we extend these analyses showing that in five exemplary cancer cell lines, derived from different cancer entities, all Anxs that are expressed in a given cell are translocated to the cell surface. The exposure of Anxs on the surface was - in general - not dependent on the mode of apoptosis induction in a given cell line, and all methods of apoptosis induction caused similar Anx surface exposure. Thus, externalised Anxs on apoptotic tumour cells are well in place to contribute to the immune suppressive microenvironment widely described within different solid tumours [86–88].

As discussed above, the exposed Anx core domain may suppress phagocytes and, thus, the anti-tumour immune response in a redundant manner [7] and the accumulated effect of all Anxs expressed has to be taken into consideration. Consequently, the total expression of all Anx family members was investigated using expression data from a publicly available online database, which allowed analysis of paired data sets of healthy and malignant tissue. These data indicated a heterogeneous situation, where total Anx expression after transformation was not consistently increased but depended on the cancer entity. The comparison of total Anx expression between adjacent healthy and malignant tissue of 7 cancer entities showed reduced expression after transformation in prostate carcinoma, breast cancer, oesophagus carcinoma and NSCLC. CRC showed no difference in Anx expression. In contrast, in PDAC and RCC total Anx expression was significantly increased in malignant samples compared to healthy tissues.

Our hypothesis of the tumour promoting influence of the Anx checkpoint system, however, depends critically on the sensitivity of the tumour towards immune mediated rejection. Thus, the immune status of the investigated tumour entities was included in the analysis. In this regard, pan-cancer immune infiltration analyses have demonstrated that the two cancer entities showing significantly increased Anx expression upon transformation, PDAC and RCC, show a more inflamed microenvironment with a higher T cell infiltration signature than other cancers [89, 90]. In contrast, Prostate and oesophageal carcinomas which downregulated Anx expression displayed low infiltration of T cells [89, 90]. For CRC, which showed no difference in regulation of Anx expression, an intermediate infiltration is described. Therefore, our results show that a higher immune infiltration in the tumour is correlated with increased total Anx expression, whereas lower infiltration correlates with decreased Anx expression. If tumours are highly infiltrated with immune cells that exert a strong selective pressure on cancer cells, upregulation of Anxs could be an immune-evasive measure. Interestingly, certain tumour entities, which show increased Annexin expression correlated with immune infiltration as discussed above, also show a correlation of high Annexin receptor (Dectin-1/CLEC7A) expression [14] with worse prognosis. This might indicate that tumours expressing high amounts of Annexin as well as the tolerogenic Annexin receptor cannot be controlled by the immune system efficiently. It will be very interesting to analyse potential functional dependencies of these features in future studies.

The only exception from the correlation of immune infiltration in the tumour and increased total Anx tumour expression is represented by data of NSCLC patients (non-smoking female patients, mostly adenocarcinoma), which are coherently reported to have high immune cell infiltration but show decreased overall Anx expression [89, 90]. Immune cell infiltration, however, is known to depend on various additional factors such as the density and presentation of neoantigens or the status of the tumour vasculature [91, 92]. Therefore, the correlation between immune cell infiltration and increased total Anx expression requires additional data from more patients, as well as other cancer entities to be confirmed. In addition, the absolute amount of Anx on the surface required to influence the immune response remains unknown. It is well possible that even low expression of few Anxs suffices to trigger a tolerogenic effect. Ultimately, engraftment experiments in syngeneic immunocompetent hosts using complete Anx knock-out tumour cell lines in comparison to Anx-overexpressing cells need to be performed to address this question. However, due to the expression of at least 5 different Anxs in any given cell line investigated so far, including rodent cell lines, such experiments appear not to be within reach at present.

Taken together, this study is the first comprehensive analysis of the expression and translocation of all Anx family members in a tumour setting. Further investigations are required, however, to validate a suppressive or tolerogenic influence of the Anx checkpoint system on the anti-tumour immune response. If so, blocking Anx-mediated immunomodulation might pose an attractive target for future therapeutic interventions.

## Conclusions

On the surface of apoptotic cancer cells, Anxs are expected to induce tolerance towards tumour antigens and inhibit tumour rejection. Tolerogenic signalling of Anxs is mediated by the highly conserved core domain, present in all members of Anx protein family.

Therefore, we investigated the expression and externalisation of all proteins of the Anx family in different cancer entities. Expression analysis showed considerable variation in the expression of the different Anx family members between cancer-derived cell lines of diverse origins. However, AnxA1, AnxA2 and AnxA5 consistently constituted the major part of total Anx expression. Furthermore, we demonstrated that all of the expressed Anx family members translocated to the cell surface upon apoptosis induction in different cancer cell lines. Investigation of human expression data from online databases indicated a correlation between immune infiltration of the respective cancer types and overall expression level of Anxs in malignant compared to healthy tissue.

The presentation of Anxs on the surface of tumour cells as an immune-evasive mechanism, could pose an attractive target for future therapies. Interference with these tolerogenic signals, especially in cancer entities with a high immune infiltration that also display high Anx expression levels, could maximise cancer immunotherapy and lead to increased cancer rejection.

Supplementary information is available at the British Journal of Cancer’s website

## Supplementary Information

**Additional file 1.** Supplementary Information Table S1.

**Additional file 2.** Supplementary Information Table S2.

**Additional file 3.** Supplementary Information Table S3.

**Additional file 4.** Supplementary Information Table S4.

**Additional file 5.** Supplementary Information Table S5.

**Additional file 6: Supplementary Information Figure S1.** Minute changes of Anx mRNA expression profiles after prolonged culture. The mRNA expression levels of all Anx family members (A1 – A13) in all cell lines were detected *via* RT-qPCR and depicted relative to Annexin A1 (bar diagrams). The mRNA Anx expression profiles remained stable in all tested cell lines over several months of culture. Time period of culture is indicated below arrows. Results represent mean ± s.d. of technical triplicates.

**Additional file 7: Supplementary Information Figure S2.** EDTA-washes of apoptotic cells suppress human DC/Monocyte activation. (A) Human monocyte-derived DCs were incubated with EDTA surface wash for 4 h. After pre-incubation, DCs were stimulated over night with the TLR agonist LPS. TNFα concentrations in the supernatant were analysed by ELISA 16 to 24 h after stimulation. (B) Representative single experiment. DCs were incubated with 50 and 100 μl of EDTA surface wash, respectively, from 2 day-aged apoptotic neutrophils. (C) Summary of independent experiments (*n*=8) depicting TNFα secretion normalised to stimulation. Results represent the mean ± s.d. measured in triplicates. (D) Human MonoMac6 cells [93] were pre-incubated with 50 μl of EDTA surface wash from untreated (live) or Etoposide-treated (apopt.) 624.38 Mel cells for 4 h. Subsequently, MonoMac6 cells were stimulated with 1 μg/ml R-848 and 20 ng/ml Phorbol-12-myristat-13-acetate (PMA). Results are representative for *n*=3 independent experiments. nd= not detectable. * *p*<0.05, ** *p*<0.01, *** *p*<0.001, **** *p*<0.0001 (unpaired, two-tailed t-test).

**Additional file 8: Supplementary Information Figure S3.** Flow cytometric cell death analysis of cancer cell lines before and after apoptosis induction. The rate of apoptotic cell death in aged human neutrophils and cancer cell lines has been analysed *via* flow cytometry using a combined 7AAD and AnxA5 staining. In neutrophils, apoptosis occurred physiologically after 2 days of ex vivo culture. Apoptosis in cell lines was induced by treatment with Etoposide, Dimethylfumarate (DMF) or UV-C radiation followed by incubation for 48 h. Untreated live cells served as controls. PE – Phycoerythrin, FITC - Fluorescein isothiocyanate.

**Additional file 9: Supplementary Information Figure S4.** Detection of Anx family members via immunoblot in lysates of the cell lines Huh-7, DU145, PC-3, HCT-116, HCT 15 and MDA-MB-231. Unedited immunoblots of all Anx family members and Tubulin detected in 50 μg total protein per lane. Lane 1: Huh-7, Lane 2: irrelevant control protein, Lane 3: DU145, Lane 4: PC-3, Lane 5: HCT-116, Lane 6: HCT 15, Lane 7: MDA-MB-231. Arrows indicate expected protein position and dotted boxes indicate the cropped part used in the main figure. Molecular weight of the marker indicated in kDa on the left.

**Additional file 10: Supplementary Information Figure S5.** Detection of Anx family members via immunoblot in lysates of the cell lines PANC-1, MIA PaCa-2, A375 and 624.38 Mel. Unedited immunoblots of all Anx family members and Tubulin detected in 50 μg total protein per lane. Lane 1: PANC-1, Lane 2: MIA PaCa-2, Lane 3: empty, Lane 4: A375, Lane 5: 624.38 Mel. Arrows indicate expected protein position and dotted boxes indicate the cropped part used in the main figure. Molecular weight of the marker indicated in kDa on the left.

**Additional file 11: Supplementary Information Figure S6.** Detection of Anx family members via immunoblot in lysates of the cell lines CaCo-2, COLO 205, SW480 and HT-29. Unedited immunoblots of all Anx family members and Tubulin detected in 50 μg total protein per lane. Lane 1: CaCo-2, Lane 2: COLO 205, Lane 3: SW480, Lane 4: HT-29. Arrows indicate expected protein position and dotted boxes indicate the cropped part used in the main figure. Molecular weight of the marker indicated in kDa on the left.

**Additional file 12: Supplementary Information Figure S7.** Detection of Anx family members via immunoblot in EDTA surface washes of the cell lines HCT 15 and PC-3 before and after apoptosis induction. Annexin family members (AnxA1-A13) were detected in the EDTA washes of different cancer cell lines before and after apoptosis induction. Apoptosis in cells was induced by treatment with Dimethylfumarate (DMF) followed by incubation for 48 h. Subsequently, the cell surface was washed with 20 mM EDTA/PBS and induction of apoptosis was confirmed by AnxA5/7AAD staining. Detection of cytosolic Erk1/2 was used as a control for cell membrane integrity in the samples. Untreated live cells served as controls. Lane 1: HCT 15 untreated, Lane 2: HCT 15 DMF-treated, Lane 3: PC-3 untreated, Lane 4: PC-3 DMF-Treated. Arrows indicate expected protein position and dotted boxes indicate the cropped part used in the main figure. Molecular weight of the marker indicated in kDa on the left.

**Additional file 13: Supplementary Information Figure S8.** Detection of Anx family members via immunoblot in EDTA surface washes of the cell line MDA-MB-231 before and after apoptosis induction. Annexin family members (AnxA1-A13) were detected in the EDTA washes of the cell line MDA-MB-231 before and after apoptosis induction. Apoptosis in cells was induced by treatment with Dimethylfumarate (DMF) followed by incubation for 48 h. Subsequently, the cell surface was washed with 20 mM EDTA/PBS and induction of apoptosis was confirmed by AnxA5/7AAD staining. Detection of cytosolic Erk1/2 was used as a control for cell membrane integrity in the samples. Untreated live cells served as controls. Lane 1: MDA-MB-231 untreated, Lane 2 MDA-MB-231 DMF-treated. Arrows indicate expected protein position and dotted boxes indicate the cropped part used in the main figure. Molecular weight of the marker indicated in kDa on the left.

**Additional file 14: Supplementary Information Figure S9.** Detection of Anx family members via immunoblot in EDTA surface washes of the cell lines Huh-7 and 624.38 Mel before and after apoptosis induction. Annexin family members (AnxA1-A13) were detected in the EDTA washes of different cancer cell lines before and after apoptosis induction. Apoptosis in cells was induced by treatment with Etoposide or UV-C radiation followed by incubation for 48 h. Subsequently, the cell surface was washed with 20 mM EDTA/PBS and induction of apoptosis was confirmed by AnxA5/7AAD staining. Detection of cytosolic Erk1/2 was used as a control for cell membrane integrity in the samples. Untreated live cells served as controls. Lane 1: Huh-7 untreated, Lane 2 Huh-7 Etoposide-treated, Lane 3: 624.38 Mel untreated, Lane 4: 624.38 Mel UV-C-treated, Lane 5: 624.38 Mel Etoposide-treated. Arrows indicate expected protein position and dotted boxes indicate the cropped part used in the main figure. Molecular weight of the marker indicated in kDa on the left.

## Data Availability

The datasets analysed during the current study are available in the Gene Expression Omnibus repository (https://www.ncbi.nlm.nih.gov/geo/). Identifiers to the specific datasets can be found in Supplementary Information Table S5.

## Abbreviations

AC: Apoptotic cell
Anx: Annexin
CRC: Colorectal carcinoma
DC: Dendritic cell
DMF: Dimethylfumarate
EDTA: Ethylenediaminetetraacetic acid
Eto: Etoposide
FCS: Foetal serum albumin
GEO: Gene Expression Omnibus
LPS: Lipopolysaccharide
MΦ: Macrophage
NSCLC: Non-small cell lung carcinoma
PBS: Phosphate-buffered saline
PDAC: Pancreatic ductal-adenocarcinoma
PS: Phosphatidyl serine
RCC: Renal clear cell carcinoma
RT: Room temperature
RT-qPCR: Reverse transcriptase quantitative polymerase chain reaction
S.d: Standard deviation
TAM: Tumour-associated macrophage
TNFα: TUMOUR necrosis factor-α
