# Supplementary figures and images for: Molecular analysis of Annexin expression in cancer
Source: BMC Cancer. 2022 Sep 19;22:994. doi: 10.1186/s12885-022-10075-8 (PMC9484247; doi:10.1186/s12885-022-10075-8)

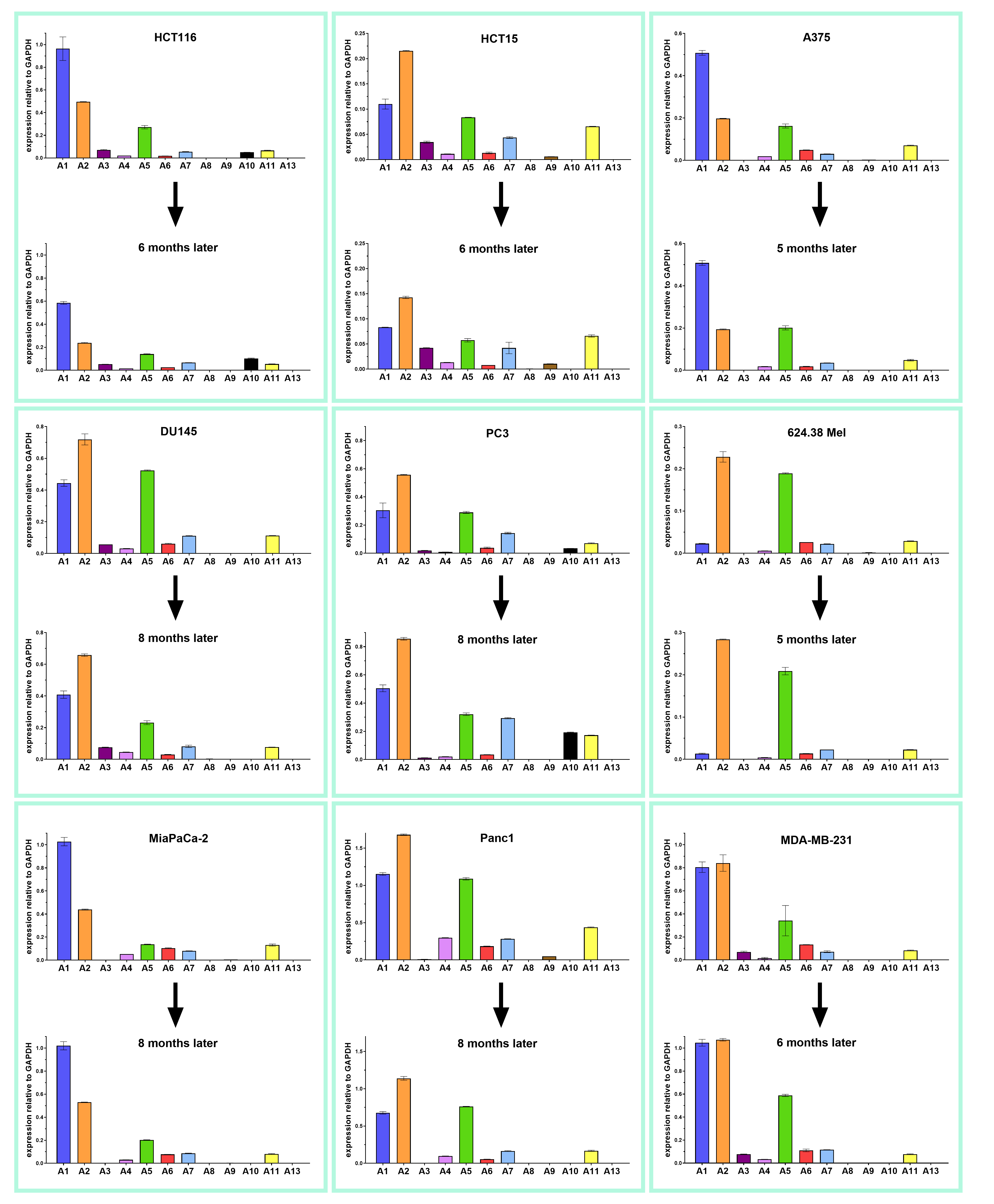

Supplement: Supplementary file 6 — Additional file 6: Supplementary Information Figure S1. Minute changes of Anx mRNA expression profiles after prolonged culture. The mRNA expression levels of all Anx family members (A1 – A13) in all cell lines were detected via RT-qPCR and depicted relative to Annexin A1 (bar diagrams). The mRNA Anx expression profiles remained stable in all tested cell lines over several months of culture. Time period of culture is indicated below arrows. Results represent mean ± s.d. of technical triplicates. [file 12885_2022_10075_MOESM6_ESM.tif]

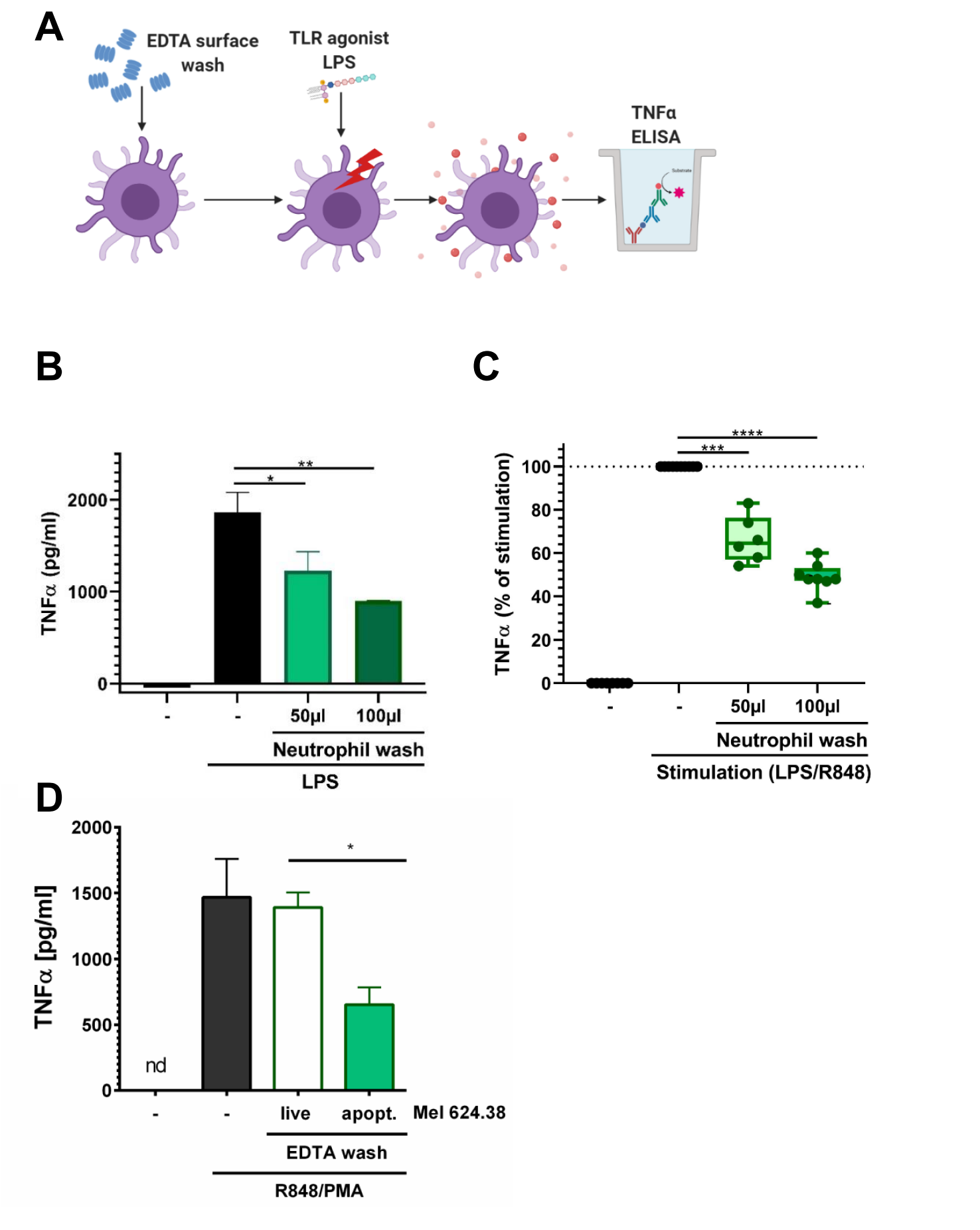

Supplement: Supplementary file 7 — Additional file 7: Supplementary Information Figure S2. EDTA-washes of apoptotic cells suppress human DC/Monocyte activation. (A) Human monocyte-derived DCs were incubated with EDTA surface wash for 4 h. After pre-incubation, DCs were stimulated over night with the TLR agonist LPS. TNFα concentrations in the supernatant were analysed by ELISA 16 to 24 h after stimulation. (B) Representative single experiment. DCs were incubated with 50 and 100 μl of EDTA surface wash, respectively, from 2 day-aged apoptotic neutrophils. (C) Summary of independent experiments (n=8) depicting TNFα secretion normalised to stimulation. Results represent the mean ± s.d. measured in triplicates. (D) Human MonoMac6 cells [93] were pre-incubated with 50 μl of EDTA surface wash from untreated (live) or Etoposide-treated (apopt.) 624.38 Mel cells for 4 h. Subsequently, MonoMac6 cells were stimulated with 1 μg/ml R-848 and 20 ng/ml Phorbol-12-myristat-13-acetate (PMA). Results are representative for n=3 independent experiments. nd= not detectable. * p<0.05, ** p<0.01, *** p<0.001, **** p<0.0001 (unpaired, two-tailed t-test). [file 12885_2022_10075_MOESM7_ESM.tif]

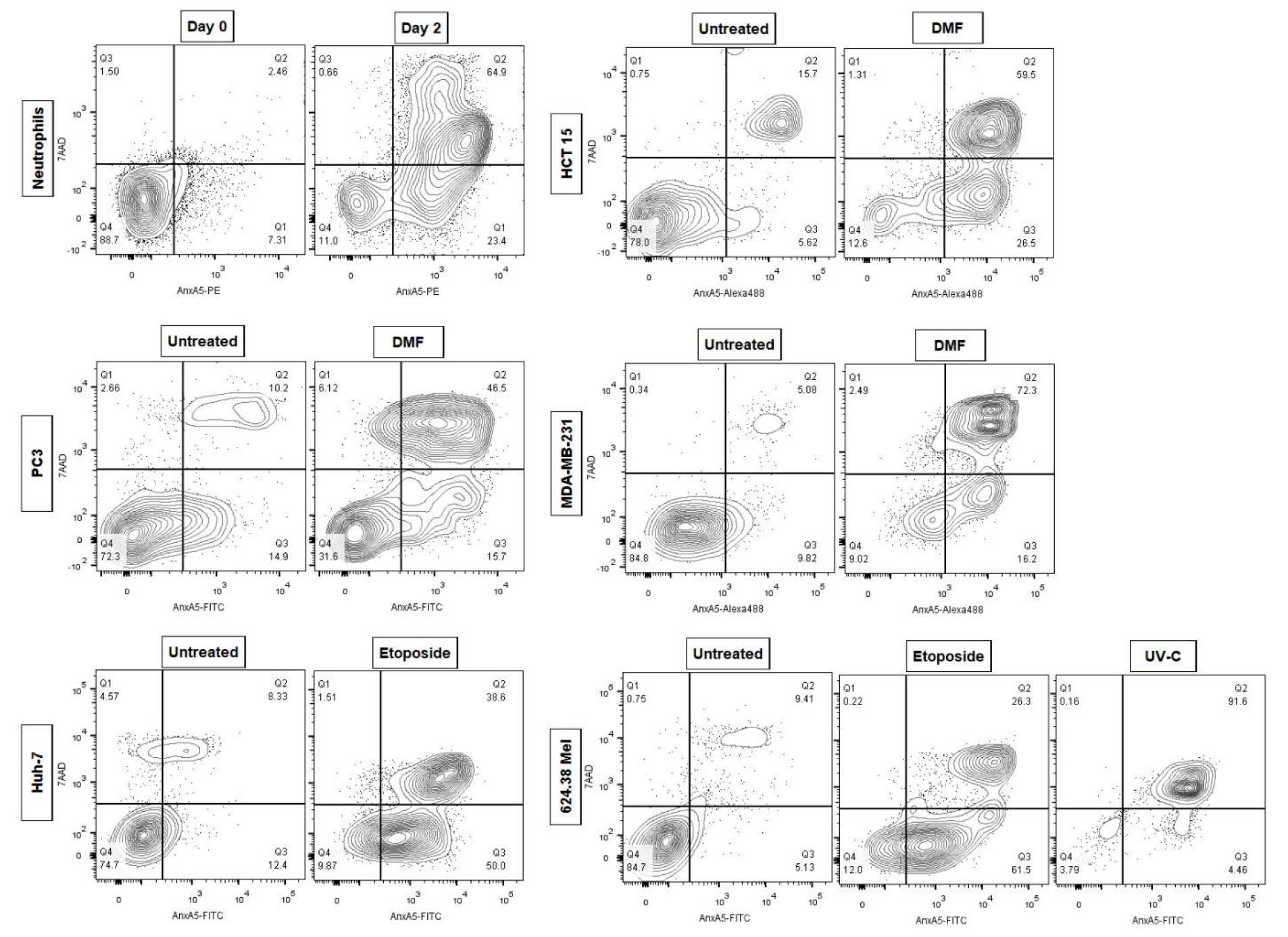

Supplement: Supplementary file 8 — Additional file 8: Supplementary Information Figure S3. Flow cytometric cell death analysis of cancer cell lines before and after apoptosis induction. The rate of apoptotic cell death in aged human neutrophils and cancer cell lines has been analysed via flow cytometry using a combined 7AAD and AnxA5 staining. In neutrophils, apoptosis occurred physiologically after 2 days of ex vivo culture. Apoptosis in cell lines was induced by treatment with Etoposide, Dimethylfumarate (DMF) or UV-C radiation followed by incubation for 48 h. Untreated live cells served as controls. PE – Phycoerythrin, FITC - Fluorescein isothiocyanate. [file 12885_2022_10075_MOESM8_ESM.tif]

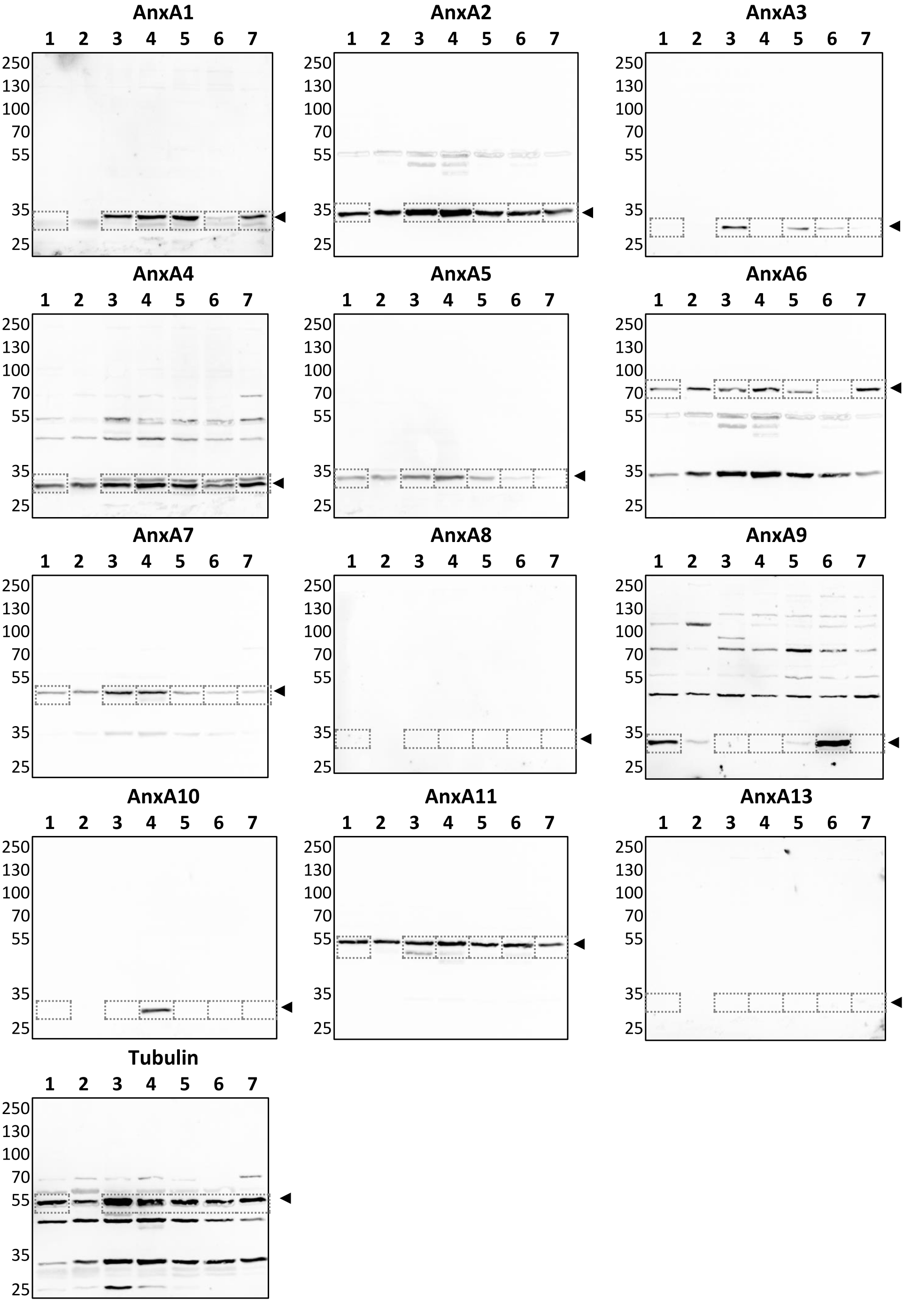

Supplement: Supplementary file 9 — Additional file 9: Supplementary Information Figure S4. Detection of Anx family members via immunoblot in lysates of the cell lines Huh-7, DU145, PC-3, HCT-116, HCT 15 and MDA-MB-231. Unedited immunoblots of all Anx family members and Tubulin detected in 50 μg total protein per lane. Lane 1: Huh-7, Lane 2: irrelevant control protein, Lane 3: DU145, Lane 4: PC-3, Lane 5: HCT-116, Lane 6: HCT 15, Lane 7: MDA-MB-231. Arrows indicate expected protein position and dotted boxes indicate the cropped part used in the main figure. Molecular weight of the marker indicated in kDa on the left. [file 12885_2022_10075_MOESM9_ESM.tif]

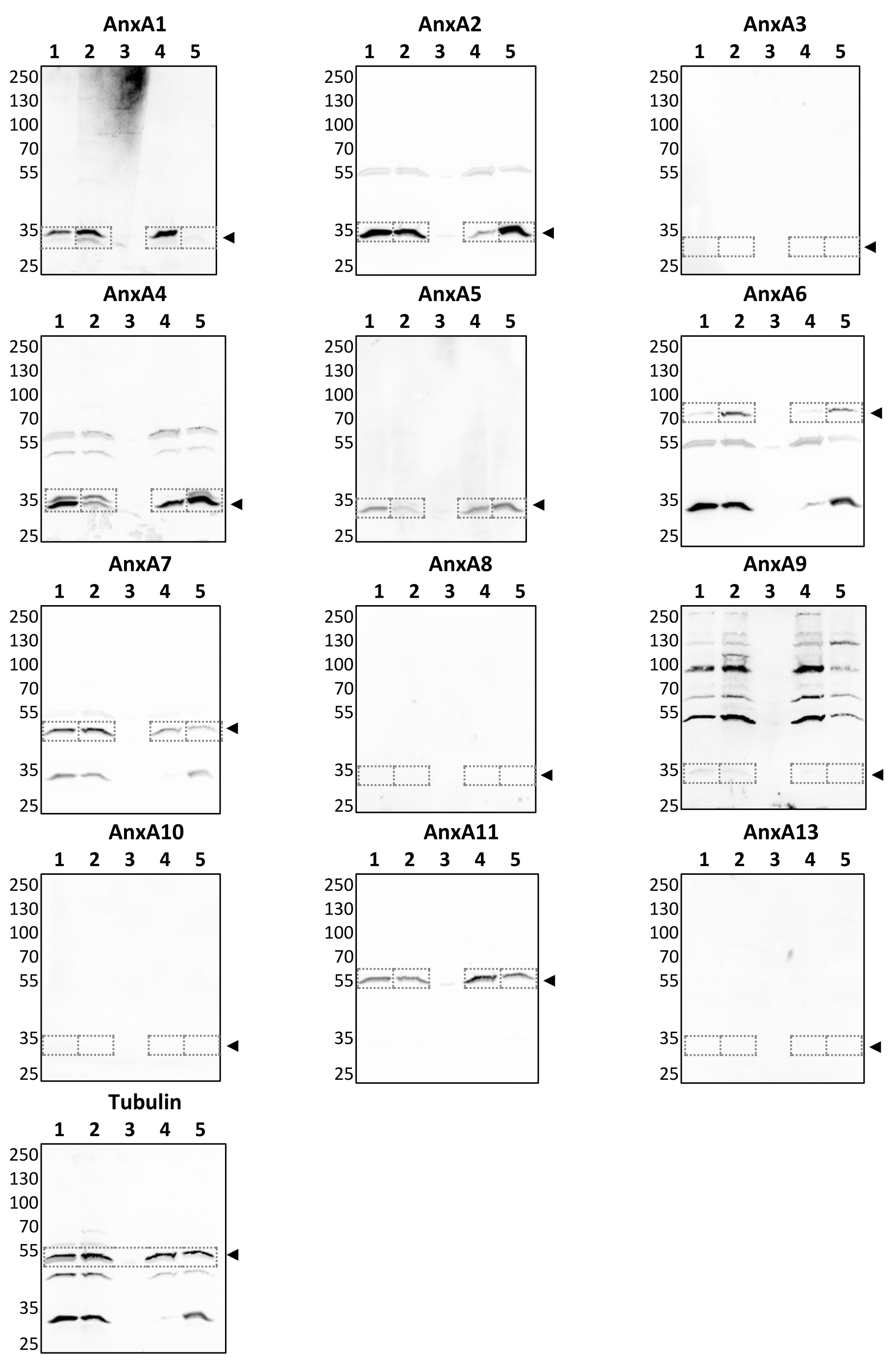

Supplement: Supplementary file 10 — Additional file 10: Supplementary Information Figure S5. Detection of Anx family members via immunoblot in lysates of the cell lines PANC-1, MIA PaCa-2, A375 and 624.38 Mel. Unedited immunoblots of all Anx family members and Tubulin detected in 50 μg total protein per lane. Lane 1: PANC-1, Lane 2: MIA PaCa-2, Lane 3: empty, Lane 4: A375, Lane 5: 624.38 Mel. Arrows indicate expected protein position and dotted boxes indicate the cropped part used in the main figure. Molecular weight of the marker indicated in kDa on the left. [file 12885_2022_10075_MOESM10_ESM.tif]

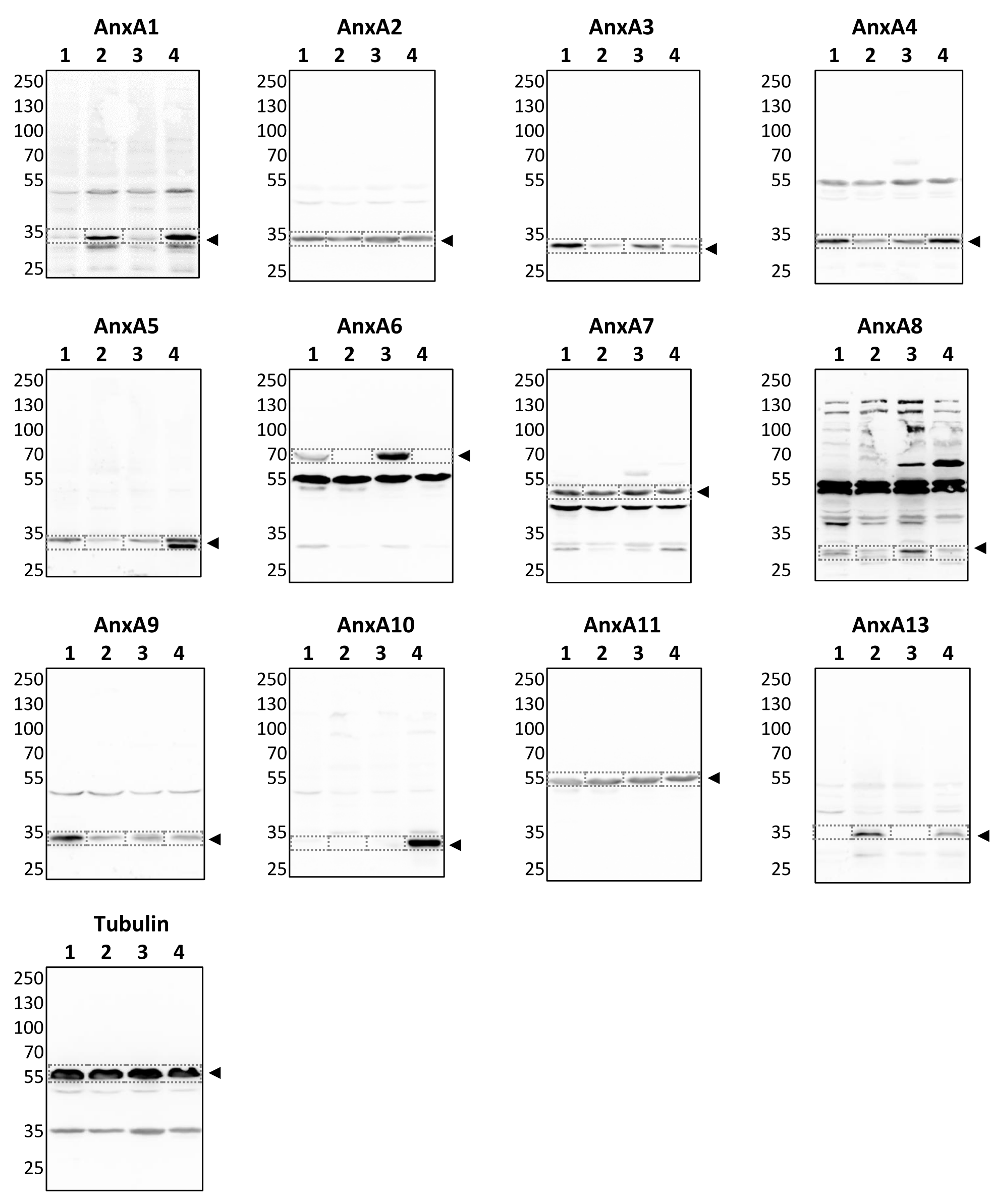

Supplement: Supplementary file 11 — Additional file 11: Supplementary Information Figure S6. Detection of Anx family members via immunoblot in lysates of the cell lines CaCo-2, COLO 205, SW480 and HT-29. Unedited immunoblots of all Anx family members and Tubulin detected in 50 μg total protein per lane. Lane 1: CaCo-2, Lane 2: COLO 205, Lane 3: SW480, Lane 4: HT-29. Arrows indicate expected protein position and dotted boxes indicate the cropped part used in the main figure. Molecular weight of the marker indicated in kDa on the left. [file 12885_2022_10075_MOESM11_ESM.tif]

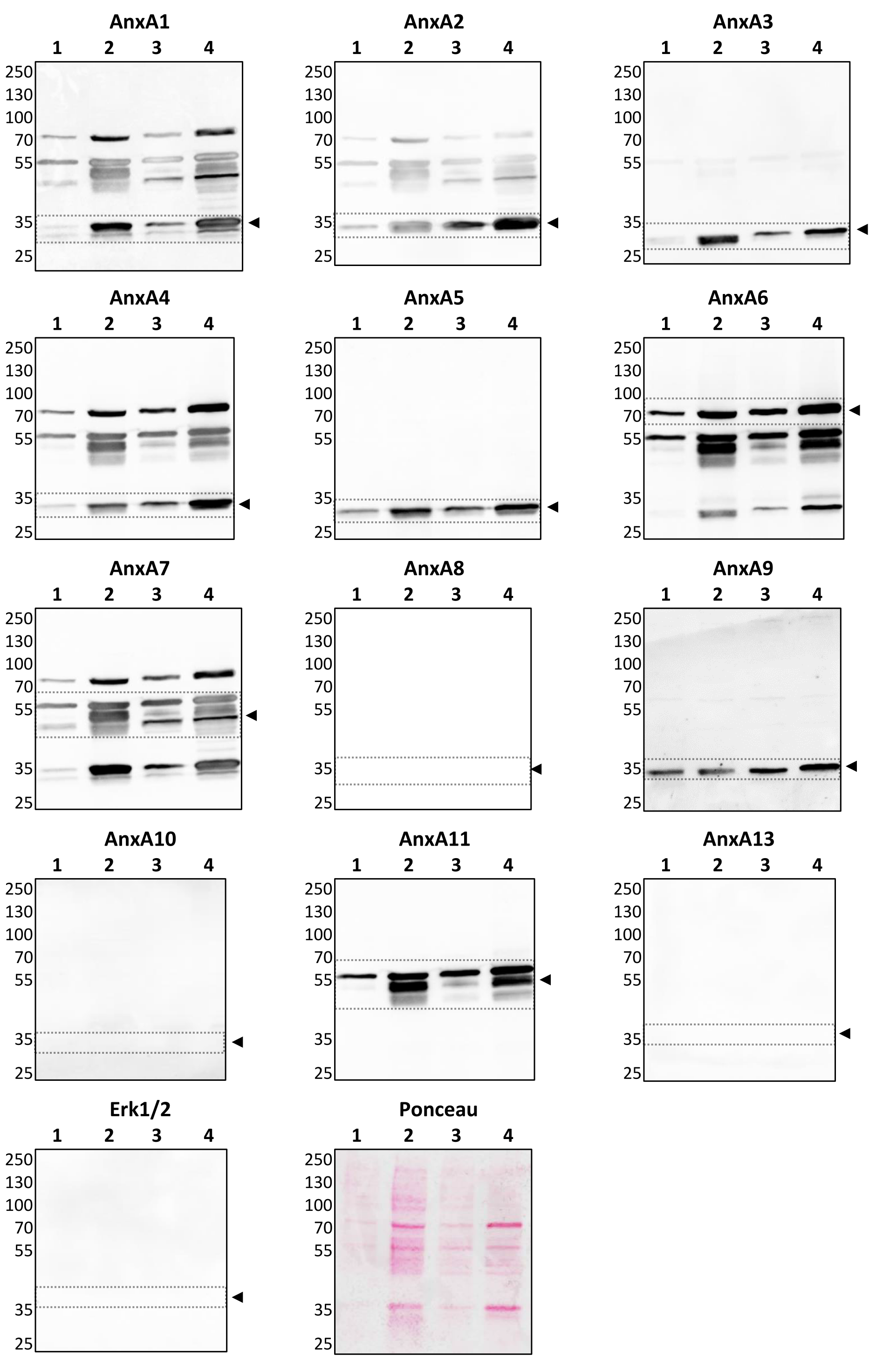

Supplement: Supplementary file 12 — Additional file 12: Supplementary Information Figure S7. Detection of Anx family members via immunoblot in EDTA surface washes of the cell lines HCT 15 and PC-3 before and after apoptosis induction. Annexin family members (AnxA1-A13) were detected in the EDTA washes of different cancer cell lines before and after apoptosis induction. Apoptosis in cells was induced by treatment with Dimethylfumarate (DMF) followed by incubation for 48 h. Subsequently, the cell surface was washed with 20 mM EDTA/PBS and induction of apoptosis was confirmed by AnxA5/7AAD staining. Detection of cytosolic Erk1/2 was used as a control for cell membrane integrity in the samples. Untreated live cells served as controls. Lane 1: HCT 15 untreated, Lane 2: HCT 15 DMF-treated, Lane 3: PC-3 untreated, Lane 4: PC-3 DMF-Treated. Arrows indicate expected protein position and dotted boxes indicate the cropped part used in the main figure. Molecular weight of the marker indicated in kDa on the left. [file 12885_2022_10075_MOESM12_ESM.tif]

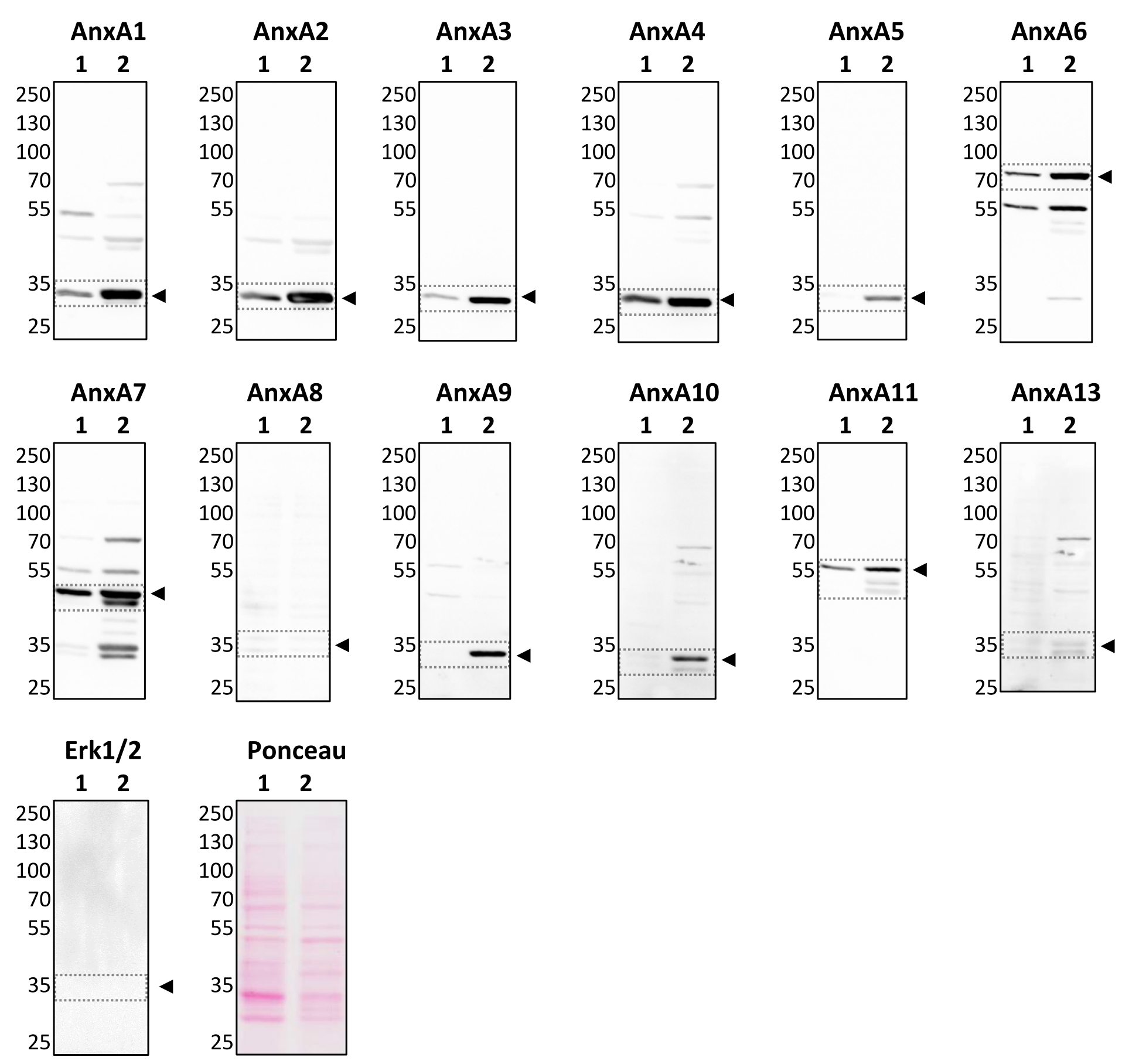

Supplement: Supplementary file 13 — Additional file 13: Supplementary Information Figure S8. Detection of Anx family members via immunoblot in EDTA surface washes of the cell line MDA-MB-231 before and after apoptosis induction. Annexin family members (AnxA1-A13) were detected in the EDTA washes of the cell line MDA-MB-231 before and after apoptosis induction. Apoptosis in cells was induced by treatment with Dimethylfumarate (DMF) followed by incubation for 48 h. Subsequently, the cell surface was washed with 20 mM EDTA/PBS and induction of apoptosis was confirmed by AnxA5/7AAD staining. Detection of cytosolic Erk1/2 was used as a control for cell membrane integrity in the samples. Untreated live cells served as controls. Lane 1: MDA-MB-231 untreated, Lane 2 MDA-MB-231 DMF-treated. Arrows indicate expected protein position and dotted boxes indicate the cropped part used in the main figure. Molecular weight of the marker indicated in kDa on the left. [file 12885_2022_10075_MOESM13_ESM.tif]

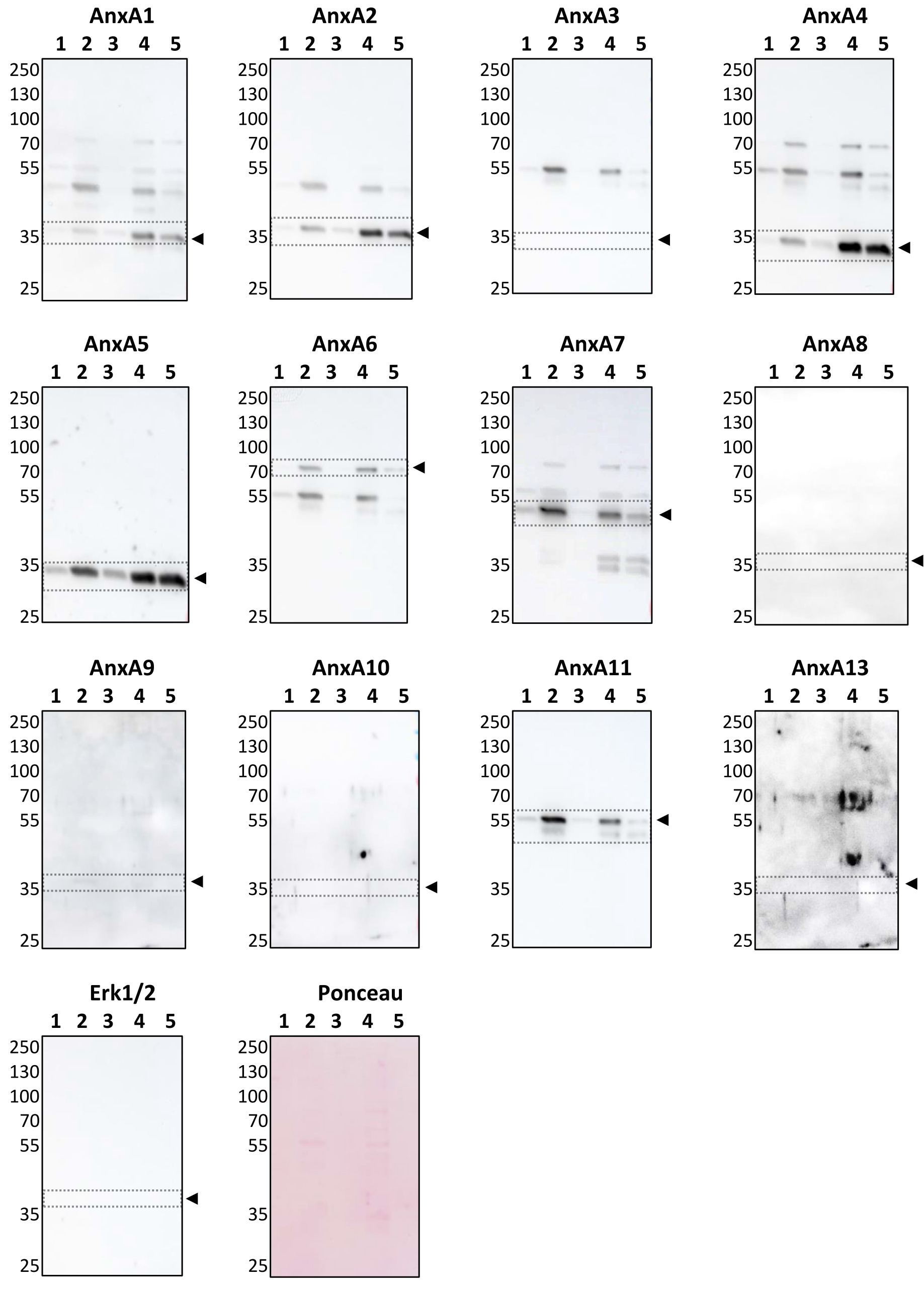

Supplement: Supplementary file 14 — Additional file 14: Supplementary Information Figure S9. Detection of Anx family members via immunoblot in EDTA surface washes of the cell lines Huh-7 and 624.38 Mel before and after apoptosis induction. Annexin family members (AnxA1-A13) were detected in the EDTA washes of different cancer cell lines before and after apoptosis induction. Apoptosis in cells was induced by treatment with Etoposide or UV-C radiation followed by incubation for 48 h. Subsequently, the cell surface was washed with 20 mM EDTA/PBS and induction of apoptosis was confirmed by AnxA5/7AAD staining. Detection of cytosolic Erk1/2 was used as a control for cell membrane integrity in the samples. Untreated live cells served as controls. Lane 1: Huh-7 untreated, Lane 2 Huh-7 Etoposide-treated, Lane 3: 624.38 Mel untreated, Lane 4: 624.38 Mel UV-C-treated, Lane 5: 624.38 Mel Etoposide-treated. Arrows indicate expected protein position and dotted boxes indicate the cropped part used in the main figure. Molecular weight of the marker indicated in kDa on the left. [file 12885_2022_10075_MOESM14_ESM.tif]
